# Disease of the Digestive Organs in Infancy and Childhood

**Published:** 1891-12

**Authors:** 


### Reviews of Books.

*Diseases of the Digestive Organs in Infancy and Childhood.* By LOUIS STARR, M.D. 2nd Edition. Pp. xi.—396. Edinburgh: YOUNG J. PENTLAND. 1891.

This is a thoroughly good book, worthy of the author's reputation. We have read it with pleasure and profit. It is clearly written, and within reasonable compass the author treats fully of the various disorders of the digestive organs. No important points fail to be considered, and the book is a model of good arrangement and concise statement. The additions to this second edition are: a section on alterations in the odour of the breath in disease; a section on urine; a chapter on massage; and a detailed account of the second dentition and its influence on the health in late childhood. The sections on treatment are throughout excellent, and we must especially commend the clearness and definiteness of the directions given for the general care and management of infants and children, and for the proper feeding of infants. Exact quantities are given in every case where feeding is discussed; nothing is left to chance, and the reader who refers to the book for help in any of the many emergencies that arise during artificial feeding, will in all cases find the information he requires in the form of clear and positive directions. Praise must also be given to the introductory remarks on the investigation of disease in children. The clinical descriptions of the different diseases are uniformly good. The book is evidently the outcome of a wide clinical experience, and from its eminently practical character may be confidently recommended.

